# Platelets and platelet extracellular vesicles in drug delivery therapy: A review of the current status and future prospects

**DOI:** 10.3389/fphar.2022.1026386

**Published:** 2022-10-18

**Authors:** Zhanqiu Dai, Tingxiao Zhao, Nan Song, Kaifeng Pan, Yang Yang, Xunbin Zhu, Pengfei Chen, Jun Zhang, Chen Xia

**Affiliations:** ^1^ Department of Spine Surgery, Zhejiang Provincial People’s Hospital, Hangzhou Medical College People’s Hospital, Hangzhou, Zhejiang, China; ^2^ Department of Orthopaedics, The Second Affiliated Hospital of Bengbu Medical College, Bengbu, Anhui, China; ^3^ Department of Pathology, Zhejiang Provincial People’s Hospital, Hangzhou, China; ^4^ Department of Orthopaedic Surgery, Sir Run Run Shaw Hospital, Medical College of Zhejiang University, Hangzhou, China; ^5^ Key Laboratory of Musculoskeletal System Degeneration and Regeneration Translational Research of Zhejiang Province, Hangzhou, China

**Keywords:** platelets, platelet extracellular vesicles, drug-loaded, targeted drug delivery, inflammation, tumors

## Abstract

Platelets are blood cells that are primarily produced by the shedding of megakaryocytes in the bone marrow. Platelets participate in a variety of physiological and pathological processes *in vivo*, including hemostasis, thrombosis, immune-inflammation, tumor progression, and metastasis. Platelets have been widely used for targeted drug delivery therapies for treating various inflammatory and tumor-related diseases. Compared to other drug-loaded treatments, drug-loaded platelets have better targeting, superior biocompatibility, and lower immunogenicity. Drug-loaded platelet therapies include platelet membrane coating, platelet engineering, and biomimetic platelets. Recent studies have indicated that platelet extracellular vesicles (PEVs) may have more advantages compared with traditional drug-loaded platelets. PEVs are the most abundant vesicles in the blood and exhibit many of the functional characteristics of platelets. Notably, PEVs have excellent biological efficacy, which facilitates the therapeutic benefits of targeted drug delivery. This article provides a summary of platelet and PEVs biology and discusses their relationships with diseases. In addition, we describe the preparation, drug-loaded methods, and specific advantages of platelets and PEVs targeted drug delivery therapies for treating inflammation and tumors. We summarize the hot spots analysis of scientific articles on PEVs and provide a research trend, which aims to give a unique insight into the development of PEVs research focus.

## 1 Introduction

Besides erythrocytes, platelets are the most prevalent and indispensable blood component, which play a key role in human blood function (Semple et al., 2011). Platelets are anucleate cells that are crucial mediators of haemostasis, with the ability to target vascular injury sites and maintain the integrity of blood circulation (Holinstat, 2017). In addition, their specializations include activities and intercellular interactions that make them key effectors in inflammation and in the continuum of immunity and tumor (Vieira-de-Abreu et al., 2012). Developments in the field of platelets biology have led to new insights into platelet formation, function, heterogeneity, and communication (van der Meijden and Heemskerk, 2019). With the rapid expansion of the field of targeted drug delivery, nanotechnology has significantly contributed to the development of drug delivery systems (Sengupta, 2017; van der Meel et al., 2019). In particular, lipid-based nanocarriers provide versatile delivery systems for drug encapsulation. Related researchers have demonstrated that platelets are immune cells with an inherent affinity for inflammation and tumor which can be used for anti-inflammatory and anti-neoplastic drug delivery (Hansson et al., 2015; Zaldivia et al., 2017; Song et al., 2019). In addition to platelets, platelets extracellular vesicles (PEVs) are also popular drug delivery platforms (Vader et al., 2016; Elsharkasy et al., 2020). PEVs are a heterogeneous group of small, lipid-bound nanoparticles that play a key role in mediating pathological and physiological processes (Tao et al., 2017; S et al., 2013; Moller and Lobb, 2020). Upon activation, platelets can release two main types of PEVs into the bloodstream, which comprising microparticles (MPs) and exosomes (EXOs) (Heijnen et al., 1999). These PEVs are different in many characteristics, including size, origins, and protein composition (Spakova et al., 2021). For example, MPs are platelet-derived larger vesicles (100 nm–1 μm) released from platelets and megakaryocytes during stress conditions, including activation and apoptosis, with a typical immunophenotype of platelets and megakaryocytes (Italiano et al., 2010; Jy et al., 2010). While EXOs with nanometer diameters (30–100 nm) come from multivesicular bodies and α-granules by the endocytic pathway, which including proteins, mRNAs, and miRNAs (Raposo and Stoorvogel, 2013). In majority studies, researchers refer to MPs and EXOs consistently as PEVs (Zeller Meidell et al., 2017). Recent studies showed that, PEVs have been found to participate in a variety of biological processes *via* intercellular and intracellular communication (Risitano et al., 2012; Laffont et al., 2013; Semple, 2013), including clotting (Midura et al., 2016), angiogenesis (Mause et al., 2010), inflammation (Boilard et al., 2010), and tumor progression (Cocucci et al., 2009; Helley et al., 2009). Moreover, these studies have suggested that PEVs hold great potential as carriers for biomarkers and therapeutic agents (Yanez-Mo et al., 2015). Therefore, PEVs are increasingly attracting the interest of researchers as biocompatible drug carriers (Han et al., 2018; Lu et al., 2019; Park et al., 2020). The purpose of this review is to summarize the role of platelets and PEVs in biological function, preparation, drug-loaded methods, and drug delivery therapies for treating diseases, with a focus on their role in inflammation and tumors. In addition, we discuss the hot spots analysis of scientific articles on PEVs and provide a research trend, which hopes to give a unique insight into the development of PEVs research focus for clinical researcher in this field.

## 2 Platelets: Source, functions, and drug delivery therapy

### 2.1 Source of platelets

Platelets are disc-shaped anucleate cells with a diameter of approximately 2 µm and are produced by the cytoplasmic lysis of mature megakaryocytes in the bone marrow through several steps (Pease, 1956). First, multifunctional hematopoietic stem cells differentiate into polyploid megakaryocytes with a diameter of 50–100 μm in the bone marrow or lungs, which mature *via* the accumulation of many proteins and membrane structures (Machlus and Italiano, 2013). Subsequently, depressions form across the surface of mature megakaryocyte membranes and protrude into the cytoplasm, forming many slender branches that ultimately fuse, resulting in cell fragmentation; these fragments are platelet precursors (Machlus et al., 2014). Sloughed platelet precursors are then released into the bone marrow sinusoids where they extend into sinusoidal vessels driven by cytoskeletal rearrangements and subsequently divide to produce platelets that enter blood circulation (Ghoshal and Bhattacharyya, 2014). Mature platelets survive in human blood circulation for 7–10 days (1–3 days in mice). The platelets are then cleared through the spleen and liver (Quach et al., 2018). Therefore, a large number of fresh platelets are renewed every day to maintain normal physiological functions in the body. Under physiological conditions, the platelet count in the human body is approximately 150 × 10^9^–400 × 10^9^ platelets/L (Semple et al., 2011). The short lifespan and regular replenishment of platelets can prevent their excessive accumulation in the body while maintaining their biological functional activity (Lu et al., 2019).

### 2.2 Functions of platelets

**FIGURE 1:**
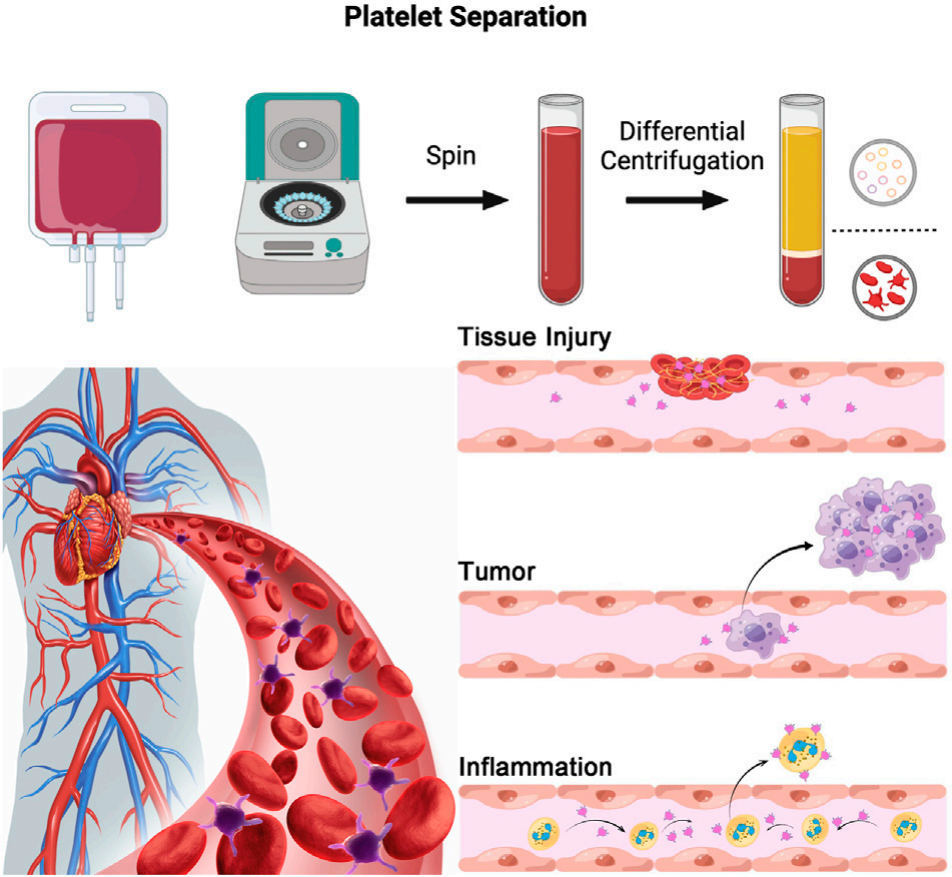
Flow chart of the isolation of platelets from whole blood. Illustration of platelets participation in physiological and pathological processes, including tissue injury, tumor development and metastasis, and inflammation.

Platelets are involved in various physiological and pathological processes, the most well-known of which is the promotion of coagulation. When a blood vessel is damaged, platelets are activated; they aggregate and adhere to the subendothelial injury to facilitate the coagulation process (Holinstat, 2017). In addition to promoting blood coagulation, platelets contain a variety of growth factors, chemokines, and cytokines, which can promote angiogenesis and regulate the recruitment, proliferation, and differentiation of cells (Theofilis et al., 2022). Embedding platelets and platelet lysates into hydrogels or other scaffolds has been studied in the context of promoting local wound healing and achieving sustained delivery of beneficial growth factors (Zufferey et al., 2012). Platelets also play an important role in the immune-inflammatory response and tumor development. Around the site of inflammation, platelets form aggregates with leukocytes *via* P-selectin, and these aggregates have been shown to play a role in the development of immune-inflammatory responses (Franco et al., 2015; Thomas and Storey, 2015). Studies have shown that platelets are involved in chronic inflammation in atherosclerotic diseases (Bernardo et al., 2005); in this research, platelets adhere to von Willebrand factor and interact with endothelial cells, which causes leukocytes to roll and eventually accumulate around endothelial cells. What’s more, Phipps (2000) also found many pro-inflammatory mediators, such as thromboxane and cluster of differentiation 40 (CD40), are then targeted for transport to the site of inflammation in atherosclerosis (Nijm et al., 2005; Devi et al., 2010); this indicates that platelets can regulate the immune-inflammatory process by enhancing leukocyte recruitment to areas of inflammation. Therefore, platelets can be used as drug delivery system to treat inflammation-related diseases. Platelets are also closely associated with pathological processes of tumorigenesis and metastasis. According to previous reports, tumor cells secrete interleukin-6 (IL-6) to stimulate thrombopoietin production at the tumor site, which in turn promotes megakaryocytopoiesis and thrombopoiesis (Kim et al., 1998; Xu et al., 2018). The platelets produced during this process can exert their effector functions to regulate tumor cell growth, maintain tumor proliferation signals, resist tumor cell apoptosis, induce tumor angiogenesis, activate invasion and metastasis, evade immune detection, and eventually support tumor stem cell growth and differentiation (Bakewell et al., 2003; Palumbo and Degen, 2007; Jain et al., 2010; Labelle and Hynes, 2012). Therefore, the development of methods that specifically target the interaction between platelets and tumor cells without disturbing the normal function of platelets may provide better treatment prospects for cancer patients as well as patients with metastatic diseases (Schlesinger, 2018). Using the above-mentioned characteristics of platelets, researchers load anti-neoplastic drugs on platelets to achieve precise targeted therapy of tumors (Figure 1).

### 2.3 Platelet drug delivery therapy

With the ongoing rapid development in the field of drug delivery, the use of cells (such as platelets, neutrophils, red blood cells) as carriers for drugs has attracted increasing attention. Compared to the traditional drug loading mode, cell drug loading offers advantages in terms of biocompatibility and immunogenicity. More specifically, platelet drug loading has several unique advantages. First, platelets are dynamic cells that can exert several unique effects when activated, including shape, transformation particle release, and contraction, which can easily trigger drug release. Second, platelets exist in large numbers and are easier and quicker to purify; thus, they have a higher potential for clinical translation. Finally, platelets are adept at targeting specific inflammation/tumor areas, thereby reducing drug accumulation in non-targeted organs, such as the liver, spleen, and kidneys (Stroncek and Rebulla, 2007; Forbes and Rosenthal, 2014; Parayath and Amiji, 2017). However, platelets may have an adverse effect on thrombosis and tumor development *in vivo* (Haemmerle et al., 2018). A previous study has demonstrated that platelet activation leads to trigger drug release and changes in platelet morphology, which may affect the stability of their carrier function (Mackman, 2008; Cao et al., 2021). Besides, due to the short shelf life of platelets, clinical storage conditions need to be modified to ensure their functional integrity. These limitations have led to the development of several platelet modifications to improve their therapeutic value as drug carriers. Below, we summarized the traditional and modified methods for platelet drug delivery.

### 2.4 Traditional platelet drug delivery system

During tissue injury, the integrity of the vascular endothelium is destroyed. This leads to the aggregation of platelets to the damaged site where they adhere to the subendothelial extracellular matrix, become activated, and eventually release different granules *in vivo* (van der Meijden and Heemskerk, 2019). By exploiting the properties of platelets to dynamically release particulate matter, researchers developed a platelet-induced drug delivery system. For example, Hansen et al. encapsulated platelets in fibrinogen to form multilayer polyelectrolyte capsules. When the platelets were activated, they actively transformed and folded the fibrin network, sequentially causing the fibrin coating of the capsules to shrink and the capsules to rupture and further triggering platelet-encapsulated biotherapeutic drug release (Hansen et al., 2017). Hu et al. (2018) also developed a stem cell-platelet combination cell delivery system, in which the platelet surface was modified with anti-programmed death-ligand 1 (PD-1) antibodies (αPD-1), then the platelets were combined with hematopoietic stem cells (HSC) through a click reaction, and finally the HSC-platelet-αaPD-1 conjugate was injected into leukemic mice. Following injection into leukemic mice, the HSC-platelet-αaPD-1 conjugate were migrated to the bone marrow site and released αaPD-1 locally. This process significantly enhanced the anti-leukemia immune response, increased the number of active T cells, produced cytokines and chemokines, and ultimately prolonged the survival of the mice. Besides, some research teams have also improved the driving force of platelets by adjusting platelet structure to better exert therapeutic effects in specific sites. Tang et al. (2020) developed a motor-like platelet micro-actuator by asymmetrically immobilizing urease on the surface of native platelets. The platelet micromanipulator retained the intrinsic targeting potential to cancer cells and pathogens during this process. Moreover, the motor-like platelet micro-actuator can produce achieved self-propulsion through the action of biofuels and targeting potential. After entering blood circulation, the platelet micro-actuator catalyzed the asymmetric decomposition of urea, ammonia, and asymmetric production of carbon dioxide. The catalytic reaction created a concentration gradient that drove migration forward *via* negative chemotaxis. This cell-based micromotor strategy enables the use of cellular carriers with an external driving force, which can satisfy additional delivery requirements, such as penetration into solid tumors or across biological barriers in the brain and gut (Esteban-Fernandez de Avila et al., 2018; Srivastava et al., 2019; Shi, 2020). There are also teams that use the characteristics of platelets involved in tumor formation to achieve tumor-targeted therapy strategies by loading anti-neoplastic drugs. Li et al. (2016) loaded interferon-γ-induced protein 10 (IP10) into platelets *via* endocytosis and found that most of the loaded proteins were in the *α* granules of platelets, forming platelet-IP10 complexes. The complexes reached the ruptured blood vessels of melanoma tissue and formed platelets microaggregates. Due to the overexpression of thrombin in cancer tissue, platelets microaggregates were activated to release the IP10-containing alpha granules, leading to the subsequent recruitment of effector immune cells for tumor immunotherapy. Besides, Hu et al. have showed that the release-through the implantation of a hyaluronic acid hydrogel-of CAR-T cells targeting the human chondroitin sulfate proteoglycan 4 (PG4), polymer nanoparticles encapsulating the cytokine interleukin-15 (IL-15) and platelets conjugated with the checkpoint inhibitor programmed death-ligand 1 (PD-L1) into the tumor cavity of mice with excised subcutaneous melanoma tumor inhibits the local recurrence of the tumor as well as the growth of distant tumors (Hu et al., 2021).

### 2.5 Modified platelet drug loading systems

#### 2.5.1 Platelet membrane coating

**FIGURE 2:**
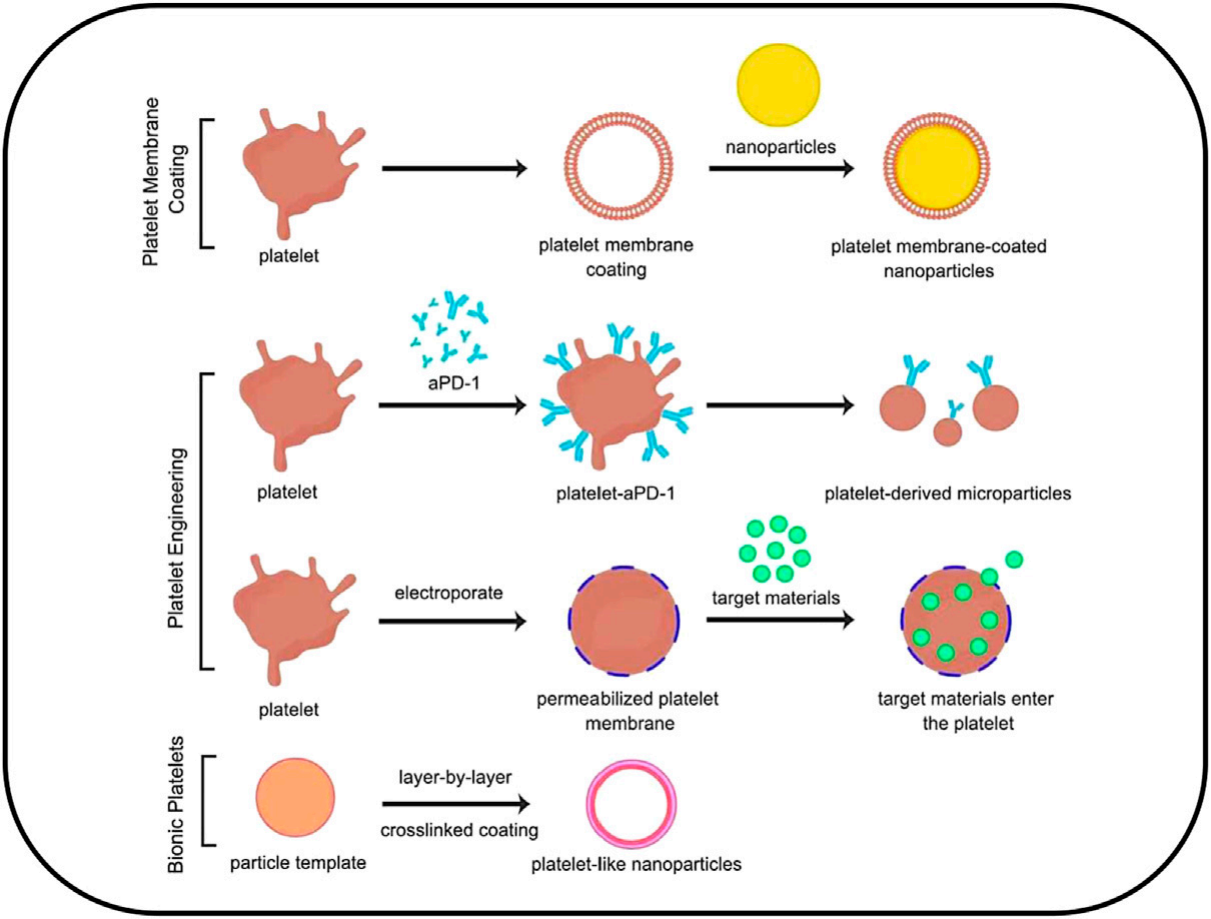
Platelet-based drug delivery systems could be categorized into three types: 1) platelet membrane coating; 2) platelet engineering, including platelet hitchhiking and electroporation; 3) Biomimetic platelets.

Platelet membrane coating is a modified method that involves wrapping platelet membrane around nanodrug particles. This is commonly achieved by the sonification of platelet membrane together with solid nanoparticles, after which platelet membrane proteins are enriched on the surface of the nanoparticles (Hu C. M. et al., 2015); this process has been reported to increase the particle size of the drug by approximately 15 nm. Compared with the original drug particles, platelet membrane coating can significantly prolong the circulation time of the drug *in vivo*, with a half-life of more than 24 h, further targeting tissues and enhancing drug accumulation (Hu et al., 2011; Hu Q. et al., 2015). This is facilitated by the “anti-phagocytosis” signal issued by the platelet surface membrane protein CD47, which reduces the phagocytosis of nano drugs by macrophages (Wang et al., 2019). In the process of tumor treatment, platelet membrane-coated nanoparticles drug can reach artificial-induced tumor vascular damage area to play a therapeutic role and implement a “relay drug delivery” strategy, thereby improving anti-tumor efficacy (Hu et al., 2017). This may provide rationale for the development of platelet membrane-coated drugs for treating other diseases as well (Figure 2).

#### 2.5.2 Platelet engineering

Platelet engineering is a method of modifying platelets using chemical, physical, biological, and other methods to connect platelets with nanomedicines. Owing to the presence of many primary amine or thiol residues on the platelet membrane surface, these special structures can chemically link platelets to nanoparticles or biomolecules (Wang et al., 2017; Li et al., 2021). For example, in the study by Hu et al. (2018) which we discussed above, the engineering of HSC-platelet-αPD-1 conjugates was used to achieve a therapeutic effect. In some cases, platelet editing can be performed by electroporation, a method that creates pores in the cell membrane and allows small molecules to enter the cytoplasm (Yarmush et al., 2014). For example, the targeting properties of platelets could be used to deliver the nanorods to the tumor site. Gold nanorods have already been used to be loaded into platelets by electroporation. In this process, gold nanorods were used to treat squamous cell carcinoma *via* light heat treatment, whereby the nanorods convert light energy into heat energy under the irradiation of an external light source (Rao et al., 2018). This injures the tumor cells, which leads to the further recruitment of platelet-nanoparticles from the circulation, thereby realizing a “self-enhancing” platelet-based cell therapy (Zeller Meidell et al., 2017). In addition, some studies also encapsulated fluorescent beads, viruses, and other particles in engineered platelets to achieve targeted drug delivery *via* platelet-targeting properties (Sarkar et al., 2013; Li et al., 2016; Xu et al., 2017).

#### 2.5.3 Biomimetic platelets

Platelets are prone to contamination and have a short shelf life because their morphology and function are unstable at room temperature (Stroncek and Rebulla, 2007), making it difficult to meet the high clinical demand (Dolgin, 2017). Thus, much effort has gone towards the development of biomimetic platelets to replace natural platelets (Sakhavand and Shahsavari, 2015; Li et al., 2018; Wang et al., 2019). Biomimetic platelets are artificially synthesized platelets that imitate the functional characteristics of platelets using a bottom-up approach (Modery-Pawlowski et al., 2013). For example, platelet-mimicking peptides have been prepared using the layer-by-layer method with flexible capsule-like nanoparticles. These synthetic platelets are similar to natural platelets and mimic four key platelet properties: discoid morphology, mechanical flexibility, biophysically and biochemically mediated aggregation, and specific clustering of activated platelets (Anselmo et al., 2014). The surface adhesion of platelet-mimetic peptides allows for their adherence and aggregation at the site of vascular injury. In a mouse model of traumatic injury, platelet-mimetic peptides were shown to accumulate at the wound site and reduce bleeding time by 65%, effectively mimicking and improving the hemostatic function of native platelets. Biomimetic platelets have a longer shelf life and are less toxic on the body; thus, they hold great promise as a therapeutic strategy for hemostatic agents and targeted drug delivery in the future. However, considering the low biological efficacy, high immunogenicity, and tumorigenesis, biomimetic platelets have not passed the certification of medical ethics and clinical safety management and issues at present (Kunde and Wairkar, 2021; Han et al., 2022). Therefore, this method is currently not used in clinical practice.

## 3 Platelet extracellular vesicles: Characterization, preparation, and drug delivery therapy

### 3.1 Characterization of platelet extracellular vesicles

Extracellular vesicles (EVs) are a heterogeneous group of cell-derived membranous-bound structures including exosomes (EXOs) and microparticles (MPs), which originate from the endosomal system or which are shed from the plasma membrane, respectively (Heijnen et al., 1999). These EXOs and MPs express and transfer functional receptors from platelet membrane, playing key roles in activation of intracellular signaling pathways (Chakrabarti et al., 2016). In addition, these EVs can be recognized by different target cells such as endothelial cells and monocyte. They are present in biological fluids and are involved in multiple physiological and pathological processes (van Niel et al., 2022). With the development of nanoscale biomaterials, EVs have attracted considerable interest as a subcellular therapeutic approach for regenerative medicine and drug delivery. EVs are now recognized as additional mechanisms for intercellular communication that allow cells to exchange proteins, lipids, and genetic material (van Niel et al., 2018; Liu and Deng, 2022). Currently, In normal physiological condition, the majority of blood EVs are derived from platelets, platelet precursor cells, or megakaryocytes in the bone marrow (Aatonen et al., 2014; van Niel et al., 2018; Ferreira et al., 2020; Liu and Deng, 2022; van Niel et al., 2022).

Platelet extracellular vesicles (PEVs), also known as platelet dust and platelet-derived particles, are granular mixtures of membrane structures produced by platelets in response to various activating stimuli. The number of PEVs in circulation is large, accounting for approximately 70%–90% of the total blood derived EVs under physiological conditions (Ferreira et al., 2020). PEVs have a diameter range of approximately 100–250 nm; are negatively charged at the zeta potential; possess surface anionic phospholipids, cell-derived antigens, cytokines, and matrix metalloproteinases; and contain mRNA and microRNA (Johnson et al., 2021). Because most PEVs naturally originate from cells, their advantages for use in drug-loaded therapy are significant. For example, the nanoscale structural properties of PEVs allow them to circulate for longer in the human body; therefore, PEVs used for drug loading can prolong drug release. In addition, PEVs can penetrate tissue barriers, such as the synovium, lymph, and bone marrow, to achieve specific therapeutic effects (Puhm et al., 2021). It has been demonstrated that majority EVs can pass the blood-brain barrier (BBB) (Saint-Pol et al., 2020; Morales-Prieto et al., 2022), also PEVs are expected to be able to cross the blood-brain barrier. Because PEVs are derived from platelets, they possess major biological markers on the membrane surface, such as CD41 and CD62P, which mediate the targeting of PEVs to local inflammation and tumor sites (Gasecka et al., 2017). PEVs deliver proteins, lipids, nucleic acids, and other biologically active molecules to target cells to regulate cellular functions, including aging-related diseases. For example, Brill et al. (2005) proposed that PEVs induce angiogenesis by releasing VEGF, b-FGF, and PDGF and promote revascularization after myocardial ischemia-reperfusion injury in rats. Sprague et al. (2008) demonstrated for the first time that PEVs can transmit CD154, stimulate the production of antigen-specific IgG, and coordinate the formation of germinal centers by reacting with CD4^+^ T cells. Qu et al. (2020) reported that PEVs promoted megakaryocyte differentiation and thrombopoiesis through miR-1915-3p. Taken together, these studies demonstrate that PEVs are crucial for the regulation of pathophysiological disease processes. Their roles in transporting proteins, lipids, and nucleic acids make PEVs new effectors of tissue regeneration and cell function. Therefore, the preparation of PEVs in sufficient yield and elucidation of their mechanisms of action will facilitate the rapid development of PEVs-based therapeutics in clinical translational medicine.

### 3.2 Preparation of platelet extracellular vesicles

With the continuous advancement in the field of activated platelets and PEVs, researchers require the preparation of PEVs in sufficient yield. There are many similarities and overlaps between EXOs and MPs in EVs. Actually, researchers cannot completely separate or distinguish them because they were affected by isolation protocols and detection technologies in the past (Raposo and Stoorvogel, 2013). Consequently, researchers have begun to develop different types of separation and extraction methods for high-purity PEVs extraction, including differential centrifugation, immunoaffinity separation, size-exclusion chromatography (SEC), and ultrafiltration (UF) (Aatonen et al., 2014; Szatanek et al., 2015; Agrahari et al., 2019). While differential centrifugation has been widely used to isolate PEVs, other techniques have emerged as credible alternatives with pros and cons associated with each (Sidhom et al., 2020). Here, we provide brief descriptions of these methods (Table 1).

**TABLE 1:** The advantages and disadvantages of each method.

Methods	Advantages	Disadvantages
Differential centrifugation or ultracentrifugation	High yield and high purity	Long time and high complexity
Immunoaffinity Separation	High specificity and strong applicability	Low yield, low recovery, and high cost
Size-exclusion Chromatography (SEC)	Fast, scalability, and high purity	Low yield and low specificity
Ultrafiltration (UF)	Fast and high recovery rate	Limited vesicle size, long time, and low purity

#### 3.2.1 Differential centrifugation

Differential centrifugation is the most common method for isolating and purifying PEVs (Aatonen et al., 2014; Rui et al., 2021; Staubach et al., 2021; van Niel et al., 2022). This method involves multiple sequential centrifugation steps at different centrifugal forces to remove intact cells, dead cells, and cellular debris. First, platelets are activated with thrombin (1 U/ml), collagen (10 mg/ml), and cross-linked peptide (1 mg/ml) or 2 mm/L calcium gluconate solution at 37°C for 90 min to maximize platelet activation. Subsequently, the platelet concentrate is sequentially centrifuged at 4°C under the following conditions: 300 × g for 10 min, 2,000 × g for 10 min, and 10,000 × g for 30 min. After centrifugation, the supernatant is transferred to a new tube, and the pellet is discarded. Finally, the supernatant is ultracentrifuged at 100,000 × g for 70 min to pellet the PEVs (Aatonen et al., 2014). By this method, lots of researchers have isolated exosomes (EXOs) with a diameter of 40–100 nm and microparticles (MPs) with a diameter of 100–1,000 nm, which derived from platelets (Heijnen et al., 1999). However, researchers could not distinguish EXOs and MPs by differential centrifugation (Aatonen et al., 2014; Schwertz and Rondina, 2018; van Niel et al., 2022). In addition, ultracentrifugation of this method can lead to the co-isolation of non-EV components like protein aggregates and lipoproteins, leading to the disruption of EVs and resulting in variable loss rates (Spakova et al., 2021). The method is complicated and time-consuming. Therefore, differential centrifugation is typically only recommended for experiments designed for vesicle characterization such as size, as these require the extraction of high-purity PEVs.

#### 3.2.2 Immunoaffinity separation

The immunoaffinity separation method involves magnetic microbeads coated with antibodies that recognize certain markers present on the surface of the EVs, which mainly used to isolate exosomes (Aatonen et al., 2014; Yang et al., 2017; Brambilla et al., 2021). After mixing the plasma sample with the antibody-coated beads, magnetic force is applied to ensure that the beads bind to the vesicles of interest. The PEVs-bound beads are then eluted with the appropriate buffer and used for further analysis. The advantage of this method is its ability to select specific PEVs populations based on marker expression, regardless of their size. Immunoaffinity separation can be combined with other methods, such as flow cytometry, western blotting, and polymerase chain reaction, to further characterize the vesicles of interest. Multia et al. have developed a new, fast, and selective immunoaffinity chromatographic method including a methacrylate-based convective interaction media disk monolithic column, immobilized with anti-human CD61 antibody, which used to isolate CD61-containing platelet-derived extracellular vesicles (PEVs) in under 60 min (Multia et al., 2019). The NTA result showed the mean size of PEVs isolates from the anti-CD61 disk monolithic column were 174 nm (SD 60 nm). The results confirmed that the immunoaffinity separation method of small PEVs ranging from 30 to 100 nm (exosomes, rather than microparticles). However, magnetic beads can only bind a certain number of PEVs due to their limited physical surface area, which results in the loss of many vesicles during isolation and purification. Moreover, PEVs not expressing the antigen recognized by the chosen antibody can be lost during the procedure (Colao et al., 2018; Multia et al., 2019). Therefore, for increasing yields, the magnetic beads can be loaded with higher antibody amount.

#### 3.2.3 Size-exclusion chromatography

Size-exclusion chromatography (SEC) is based on the principle that particles in a sample pass through a filter column at different speeds according to their size, thereby achieving separation and purification (Aatonen et al., 2014; Ferreira et al., 2020; Sidhom et al., 2020; Grangier et al., 2021). Samples are first passed through micron-sized pore filters of different sizes to remove larger cell debris, and then, the filtered samples are passed through a gel filtration column for SEC. Larger particles elute faster, whereas smaller particles penetrate the stationary phase (gel) of the column and are retained. The filtrate is collected and ultracentrifuged at 100,000 × g for 1 h or longer to pellet the PEVs (Antich-Rossello et al., 2020). Ideally, the eluted fraction obtained at a given time should contain a set of particles of the same size. The obtained PEVs are then resuspended in phosphate buffer and can be used for downstream assays. SEC is reproducible, scalable, inexpensive, and does not require specialized equipment or user expertise. However, this methodology cannot distinguish between EXOs and MPs of the same size (Sidhom et al., 2020). Where identification of EV subtype is important to address, combining SEC with immunoaffinity separation is recommended, despite the lower yield and some loss in purity (Baranyai et al., 2015).

#### 3.2.4 Ultrafiltration

Ultrafiltration (UF) is a special technique depends on the use of membranes with specified pore diameters to isolate EVs of a pre-determined size range (Cheruvanky et al., 2007; Lobb et al., 2015). This protocol can be used as a complement to ultracentrifugation to separate large MPs and small EXOs, though it also can be used as a stand-alone technique. Larger MPs are eliminated first by utilizing filters with pore diameters of 0.8 and 0.45 µm, leaving a relatively exosome-rich filtrate. Smaller EXOs are then limited in the filtrate by using membranes with pores smaller than the desired exosomes (0.22 and 0.1 µm). The EXOs are obtained by a maximal and minimal size range *via* pore filtration membrane. The advantages of UF are that it is comparatively less time- and labor-intensive and does not require the use of expensive equipment (Konoshenko et al., 2018). However, compared with other techniques, UF has lower EVs yield and purity, with poorer quality of RNA, and microRNA (Alvarez et al., 2012). Currently, this technology is not fully applicable to the isolation of PEVs (Mareschi et al., 2022).

### 3.3 Targeted drug delivery therapy with platelet extracellular vesicles

**FIGURE 3:**
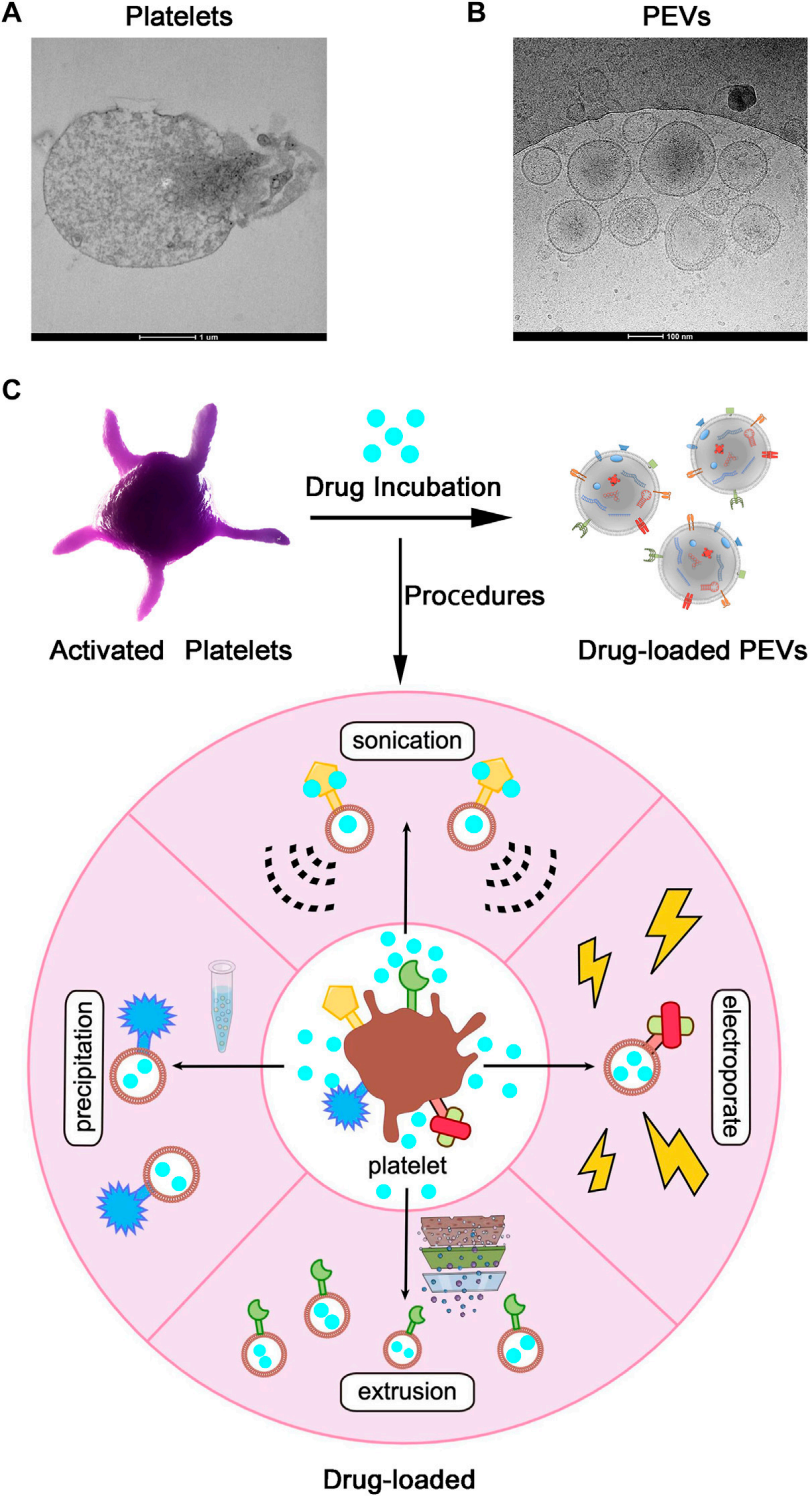
Application of Platelet Extracellular Vesicles (PEVs) as Drug-Loaded Delivery Systems. **(A)** Transmission electron microscope images of Platelets and **(B)** PEVs. Scale bar = 1 µm, 100 nm. **(C)** After activation or other processes, platelets shed PEVs, which inherit membrane properties from the parental platelets. PEVs can be loaded with drugs by the application of various procedures (such as precipitation, sonication, extrusion, electroporation, and so on) and used as targeted drug delivery systems.

In recent decades, nanotechnology has made significant contributions to the development of drug delivery systems. As we all know, PEVs are involved in major physiological and pathological processes, including cellular homeostasis, transmission of infection, cancer development, and cardiovascular disease (Herrmann et al., 2021), and PEVs offer several biological advantages (such as high stability, low immunogenicity, low tumorigenesis, and better tissue penetration) over the traditional synthetic vectors. Due to these excellent biological properties, PEVs have been applied for drug delivery systems. In theory, both hydrophobic and hydrophilic drugs can be loaded into PEVS through the outer lipid bilayer membrane (Lener et al., 2015; Walker et al., 2019). There are two main methods for loading drugs into PEVs: 1) phagocytosis by platelets and 2) direct loading into PEVs. The first method involves incubating platelets with the drug, which will result in drug phagocytosis. Subsequently, the platelets will release PEVs containing the phagocytosed drug, which can be isolated from the culture media. The second method of directly loading PEVs with drugs (rather than platelets) can be accomplished using a variety of techniques, such as incubation, sonication, electroporation, extrusion, dialysis, freeze-thaw, saponin treatment, and transfection, which are very similar to platelet drug-loading approaches (Figure 3). Through these mature methods, drugs delivered by platelet extracellular vesicles can treat different kinds of disease (Table 2).

**TABLE 2:** Drugs delivered by platelet extracellular vesicles and treatment of disease.

Platelet extracellular vesicles deliver drugs	Curing disease
Carry doxorubicin	Leukemia (Kailashiya et al., 2019)
Delivering bortezomib	Multiple myeloma (Hu et al., 2016)
Delivery of lamivudine and tenofovir	Viral diseases (Bateman et al., 2016)
Encapsulate streptokinase and release	Thrombotic disease (Pawlowski et al., 2017)
Delivery of aPD-1 antibodies	Breast, colon, and melanoma recurrence after surgery (Li et al., 2022)
Pretreatment of hematopoietic stem cells	Colitis (Yemm et al., 2015)
Deliver TPCA-1	Acute lung injury (Ma et al., 2020)

#### 3.3.1 Cancer

As natural cell membrane derivatives, PEVs can target inflammation and tumor sites, and play important roles in infection and the tumor microenvironment. Depending on the specific conditions within the tumor microenvironment, PEVs may stimulate or inhibit tumorigenesis by influencing tumor growth, metastasis, angiogenesis, and the pathological process of tumor cell multidrug resistance (Johnson et al., 2021; Puhm et al., 2021). Recent studies have demonstrated that PEVs can regulate tumorigenesis by transferring their cargo molecules between cancer cells and cells in the tumor microenvironment, which leads to tumor growth and metastasis (Labelle and Hynes, 2012); however, some researchers have reported that PEVs can also downregulate certain tumor genes to inhibit tumor growth (Saint-Pol et al., 2020; Morales-Prieto et al., 2022). For example, Michael et al. reported the transfer of platelet-derived miR-24 from PEVs into tumor cells after the infiltration of solid tumors, such as lung and colon cancers. miR-24 acted on the mitochondrial mt-Nd2 and Snora75, thereby causing mitochondrial dysfunction, cell growth inhibition, and ultimately promoting tumor cell apoptosis (Michael et al., 2017). In addition, Gasperi et al. reported that PEVs could target human breast cancer cells; when endocytosed by cancer cells, PEVs began to release miR-233, which inhibited tumor growth (Gasperi et al., 2019). In any case, PEVs play a significant role in regulating the process of tumorigenesis. Thus, more and more researchers take its properties to load drugs to further treat tumors. Previous studies have demonstrated the use of PEVs as “Trojan horses” to carry drugs for cancer (Sprague et al., 2008). Ying et al. prepared PEVs loaded with doxorubicin (Dox-PLT) or vancomycin (Vanc-PLT) and demonstrated that Dox-PEVs and Vanc-PEVs could target tumor sites, as well as sites of infection and inflammation, to deliver more drugs than drug-loaded erythrocyte membrane vesicles (Qu et al., 2020). These experimental results revealed that PEVs are highly effective in active targeted therapy. In another study, Li et al. developed a platelet-binding αPD-1 antibody for the prognosis of tumor surgery. The inflammatory environment after surgery may trigger platelet activation, leading to the secretion of PEVs containing αPD-1 antibodies and the release of αPD-1, thereby reactivating T cells for a sustained immune response against tumor recurrence. These results suggest that PEVs can act as “second carriers” to enhance the therapeutic effect of local and systemic αPD-1 antibodies on tumor recurrence after surgery (Szatanek et al., 2015). Kailashiya et al. used engineered PEVs as chemotherapeutic drug carriers for the treatment of leukemia cells (Agrahari et al., 2019). Hu et al. prepared engineered nanoplatelet-encapsulated nanocarriers to target the bone microenvironment and myeloma cells to inhibit the growth of multiple myeloma (Sidhom et al., 2020). The interest on the PEVs field arises rapidly, opening new avenues on the role of PEVs in cancer and their emerging potential as biomarkers and therapeutic agents.

#### 3.3.2 Immuno-inflammation

Besides to tumor disease, PEVs-based drug delivery systems are also applied to treat inflammation disease. As a kind of immune cells, platelets and PEVs has an inherent affinity for inflammation sites including atherosclerotic plaques site, which could be used for anti-inflammatory agents delivery (Zaldivia et al., 2017; Song et al., 2019). Like platelets, PEVs contribute to the activation of host immune cells by attracting leukocytes to areas of pathogen invasion, facilitating leukocyte-to-leukocyte interactions, and inducing the release of specific cytokines (Aatonen et al., 2012; Dinkla et al., 2016). PEVs contribute to inflammation in infection through direct recruitment of leukocytes, including T cells, B cells, and monocytes, *via* chemokine release and facilitate the interaction between monocytes and endothelial cells by binding P-selectin and PSGL1 (Nomura et al., 2000; Sibikova et al., 2018). At last, PEVs to target inflammation area for effective drug-loaded delivery system (Ma et al., 2021). PEVs can also act as extracellular traps for neutrophils to form thrombus scaffolds and lead therapeutic drug to sites of inflammation under certain pathological conditions (Jiao et al., 2020). In a mouse model of acute lung injury, it was shown that intravenous injection of PEVs loaded with the anti-inflammatory drug [5-(p-Fluorophenyl)-2-ureido]thiophene-3-carboxamide (TPCA-1) effectively targeted the lung injury, relieving the wound environment of inflammatory factors (Ma et al., 2020). In addition to contributing to our knowledge of an individual’s personal response to an Immuno-inflammation, these findings further highlight the role of PEVs as mediator of the inflammatory response to infection.

#### 3.3.3 Thrombosis

Because of high pro-thrombotic nature, PEV membranes have 50–100-fold higher procoagulant activity than activated platelets (Wolf, 1967; Dyer et al., 2019). The extent to which PEVs can increase thrombosis depends on both their number and composition. PEVs from pathologic conditions, such as disseminated intravascular coagulation (DIC), thrombocytopenia, and systemic lupus, are larger in diameter and richer in amount (Ponomareva et al., 2017; Eustes and Dayal, 2022). Terrisse et al. (2010) found that PEVs adhered to endothelial cells dependent on lactadherin, PS, and αVβ3 integrin. This interaction triggered ROS production in endothelial cells which increased the amount of VWF expressed on the endothelial cell surface resulting in clot formation. Needless to say, PEVs are a new candidate biomarker of arterial thrombosis. It can be measured in biorepositories, thereby offering the possibility to validate PEVs in multicenter clinical trials (Gasecka et al., 2017).

#### 3.3.4 Cardiology

Exosomes have demonstrated both pro-and anti-angiogenic potential depending on the cardiovascular disease (CVD) microenvironment (Alcayaga-Miranda et al., 2016; Dalirfardouei et al., 2019). Ibrahim et al. demonstrated that cardiosphere-derived cells (CDC) derived exosomes promoted angiogenesis and cardiac regeneration in scarred and infarcted hearts through activation of miRNA-146a (Ibrahim et al., 2014). The results suggest that exosomes have therapeutic potential through multiple pro-angiogenic cargos. In addition, platelets and PEVs have the potential to guide living cells towards injured tissues. Taking advantage of this feature, Tang et al. (2020) induced the fusion of cardiac stem cells and PEVs *in vitro* using through polyethylene glycol and found that the modified cardiac stem cell membranes expressed platelet surface markers. The modified cardiac stem cell can promote the transport of stem cells to the site of vascular injury and increased the number of live stem cells in infarction area, further resulting in improved repair ability and reduced infarct size (Stroncek and Rebulla, 2007). The results demonstrated that PEVs can be employed to target stem cells to the heart injury sites. Although the precise mechanisms involved in the protective role of PEVs in cardiovascular diseases is still unclear, the capacity of PEVs to carry vast biological cargos and their ability to transfer a wide array of bioactive molecules to target stem cells may explain the beneficial effect of EVs as a therapy for cardiovascular diseases (Zaldivia et al., 2017). PEVs can be used as a drug delivery system to increase solubility, stability, and bioavailability of hydrophobic drug in the blood circulation (Sun et al., 2010).

### 3.4 Exosomes’ emergence as a potential target for drug delivery

The importance of PEVs function in different diseases proves that it is an indispensable mediator of drug delivery system (Koupenova et al., 2018; Kerris et al., 2020). In addition to these targeting functions, Platelet-derived exosomes exhibit stronger permeability and retention effects than platelets owing to their nanoparticle size range of 30–100 nm, which allows them to penetrate the tumor or inflammation microenvironment and deliver anticancer or anti-inflammatory drugs to target abnormal cells more easily (Sarkar et al., 2013). The unique drug-loaded PEVs therapeutic approach demonstrates the advantages of PEVs for translational applications in disease treatment and regenerative medicine (Antich-Rossello et al., 2021). Further evaluation of the efficacy of PEVs-based drug delivery systems is crucial for the sustained development of PEVs therapeutics. Overall, the effectiveness of platelet-derived exosomes as nanoscale drug delivery system combined with the fact that they inherit the beneficial characteristics of their parental platelets has resulted in researchers’ intense interest in PEVs-based drug delivery system (Wu et al., 2021).

## 4 Future prospects for platelets and their vesicles in drug delivery systems

Owing to the unique ability to target damaged tissue, platelets are widely used in drug delivery systems to provide reliable therapeutic strategies for inflammatory diseases, cancer, and other diseases. However, due to the low permeability and low stability of platelets, the clinical efficacy of platelets-based delivery systems needs to be further improved. PEVs have excellent biocompatibility and the ability to cross cellular barriers, infiltrate tissues, target pathological sites, and remain in circulation for prolonged periods. The complex composition of bioactive molecules and unique targeting glycoproteins make PEVs more suitable for therapeutic strategies for wound healing and tissue regeneration; however, the underlying mechanisms, principles, and safety of platelet vesicle cell therapy for clinical use remain unclear. PEVs contains a large number of components including proteins, lipids, RNAs, but the effective components that play their role are still unclear. The future research needs to further focus on the specific effective components in PEVs, which better improve their biological effects. Despite the unique and personalized potential of PEVs for targeted drug delivery, preparation methods in different laboratories result in differences in the platelet vesicle structure and function. To date, PEVs have been produced with low yield and drug-loading efficiency, and information from clinical trials is very limited (Thery et al., 2018; Agrahari et al., 2019), making their translation to the clinic difficult. PEVs face many technical challenges, including the optimization of isolation and preparation methods (chromatography and centrifugation), prevention of bacterial contamination, and improvement of storage conditions, shelf life, and quality control. Thus, it is a significant milestone that researchers have improved the separation and purification technology of PEVs to standardize and industrialize their clinical use. To overcome the challenges of PEVs, Gao et al. (2017) reported a strategy that generates EVs using nitrogen cavitation, which can produce vesicles from any cell with high yield and high reliability. Nitrogen cavitation is highly scalable, offers high reproducibility, and is used for large-scale production, potential clinical translation, and personalized nanomedicine treatment. At the same time, it is important to select a suitable source of platelets, as allogeneic sources are more convenient and faster to prepare. Sung et al. (2019) reported that PEVs could be generated directly from collected platelet concentrates and that approximately 200 ml of platelet concentrate contained 4–7 × 10^11^ platelets, yielding approximately 10^13^–10^14^ PEVs. It should be noted that allogeneic platelet concentrates must meet ethical requirements, and strict regulatory measures must be established to ensure their safety. Moreover, vesicles obtained from allogeneic individuals vary greatly, and the transfusion of allogeneic PEVs may pose a risk of viral transmission. To reduce the risk of pathogen transmission, it is necessary to establish a complete virus clearance program without sacrificing PEVs function (Johnson et al., 2021). Ideally, autologous sources are best as they reduce the risk of immune rejection by the host; however, due to the time required for preparation, autologous sourcing may only be suitable for patients undergoing elective surgery. Given the effectiveness of PEVs-based targeted drug delivery thus far, further research in the field is likely to reveal new insights for overcoming remaining obstacles, yielding new effective treatment strategies for a range of diseases.

## 5 Analysis of hot spots trends in bibliometric visualization of platelet vesicles

Currently, researchers in the cell therapy and biotechnology industries are interested in the potential of PEVs. The number of scientific publications describing the physiological and pathological functions of PEVs has increased dramatically in the last decade (Thery et al., 2018); however, there is no complete visual data analysis of the preparation and drug-loaded application of PEVs. Therefore, we searched the Web of Science using the keywords “platelet extracellular vesicles,” “exosomes,” “microvesicles,” and “microparticles” to summarize and analyze the hot spots trends of platelet vesicles in the last 5 years.

**FIGURE 4:**
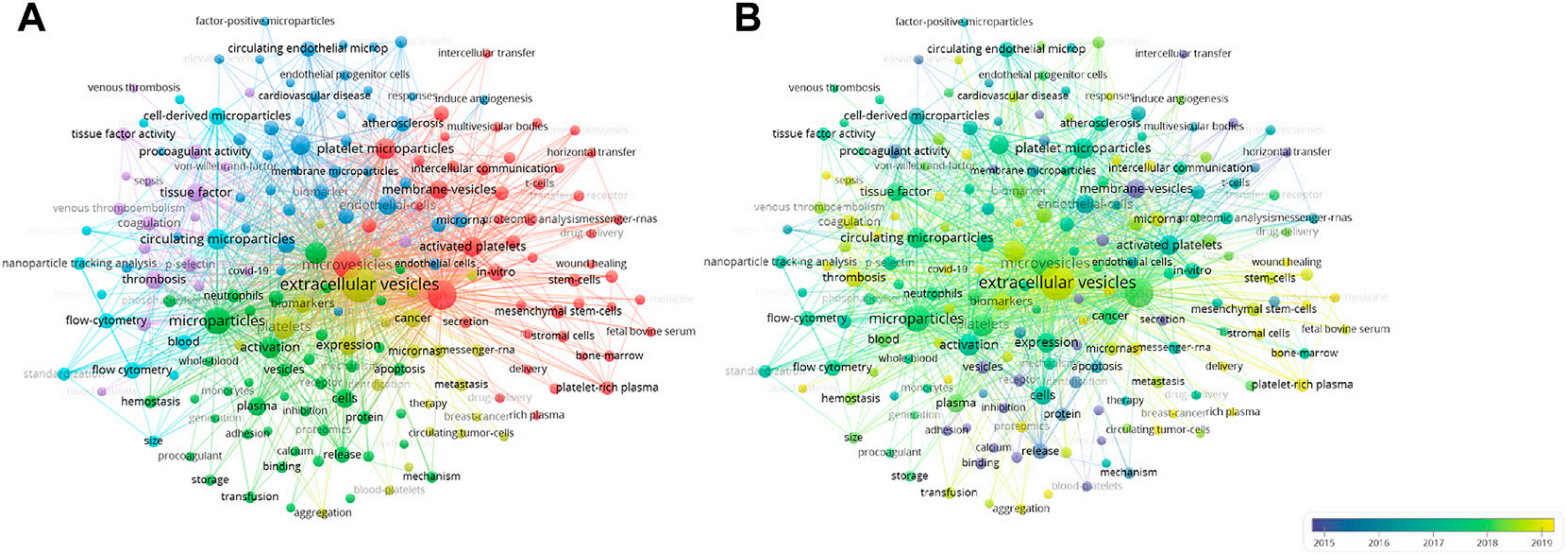
Keyword analysis. **(A)** Network visualization map showing cluster analysis of keywords associated with platelets extracellular vesicles. Different colors represent different clusters, green for “vesicle secretion mechanism” cluster, red for “biological function” cluster, blue for “methods and omics” cluster, purple for “pathophysiology” cluster, and yellow for “clinical diseases” cluster. **(B)** Network visualization map showing evolution of keyword frequency over time. Colors were assigned according to the average year in which keywords appeared in articles.

We used VOSviewer to perform hotspot analysis of publications on PEVs with a minimum of 10 occurrences. A total of 205 popular keywords were included, which were divided into five categories: “vesicle secretion mechanism,” “biological function,” “applied methods and omics,” “pathophysiology,” and “clinical diseases.” (Figure 4A) In the “vesicle secretion mechanism” curve, the most popular keywords were “microvesicle” and “activation”. The keywords “platelet extracellular vesicles” and “activated platelets” were the most common keywords in the “biological function” curve. In the “applied methods and omics” curve, the most popular keywords were “flow cytometry” and “nanoparticle tracker.” In the “pathophysiology” and “clinical disease” curves, “tissue growth factor” and “cancer metastasis” were the most popular keywords.

Additionally, to further identify dynamic change in research topic, we assessed the evolution of the most frequent keywords in the periods (Figure 4B). Colors were assigned based on the average year in which keywords appeared in articles. For instance, purple keywords appeared earlier than yellow keywords. In the early stage of PEVs research, the “membrane-vesicles” “release,” “binding,” “procoagulant activity,” and “adhesion” were the main hot spots. Recent trends showed that the words “delivery,” “diseases,” “wound healing,” “thrombosis,” and “hemostasis” increased in popularity. This analysis revealed that biological function assessment was an early focus that later shifted to clinical therapy and drug delivery. Experts studying the field of PEVs are advised to be familiar with this change in research interests. In conclusion, this visual hotspot analysis of PEVs will provide useful guidance to clinicians and researchers in the field and create market development opportunities for platelet vesicle therapeutic applications.

## 6 Conclusion

To summarize, in this article, we have discussed the characteristics of platelets and PEVs that render them useful vehicles for targeted drug delivery and have highlighted the effectiveness of such systems in the context of various diseases, including cancer. These findings demonstrate that PEVs can promote intercellular and intracellular signaling and mediate targeting to sites of inflammation and tumors for drug release. In the future, research on drug-loaded nanovesicle therapy will elucidate the mechanisms of action in different disease contexts. Overall, drug-loaded PEVs therapy has broad, promising implications for the treatment of multiple systemic diseases.
